# Visceral-to-subcutaneous fat ratio is a possible prognostic factor for type 1 endometrial cancer

**DOI:** 10.1007/s10147-021-02060-1

**Published:** 2021-10-30

**Authors:** Michiko Wada, Ken Yamaguchi, Hajime Yamakage, Takayuki Inoue, Toru Kusakabe, Kaoru Abiko, Kenji Takakura, Ikuo Konishi, Noriko Satoh-Asahara

**Affiliations:** 1 Department of Endocrinology, Metabolism, and Hypertension, Clinical Research Institute, National Hospital Organization Kyoto Medical Center, 1-1 Mukaihata-cho, Fukakusa, Fushimi-ku, Kyoto 612-8555 Japan; 2 Department of Obstetrics and Gynecology, National Hospital Organization Kyoto Medical Center, Kyoto, 612-8555 Japan; 3 Department of Gynecology and Obstetrics, Kyoto University Graduate School of Medicine, 54 Kawahara-cho, Shogoin, Sakyo-ku, Kyoto 606-8507 Japan

**Keywords:** Endometrial cancer, Visceral fat, Subcutaneous fat, Prognosis

## Abstract

**Background:**

Associations have been observed between obesity defined by the body mass index (BMI) and the incidence of endometrial cancer. However, the impact of obesity on the prognosis of endometrial cancer is not yet clear. Recently, visceral fat has been considered to have a greater impact on malignant disease in obese patients than subcutaneous fat. In this study, we investigated the association between prognostic factors of type 1 and type 2 endometrial cancer and obesity parameters.

**Methods:**

The impacts of clinical factors on the progression-free survival (PFS) and overall survival (OS) were analyzed retrospectively in 145 primary endometrial cancer patients. The factors included age, BMI, pathological findings, Federation of Gynecology and Obstetrics (FIGO) stage, status of lymph node metastasis, and the amounts of visceral and subcutaneous fat obtained from computed tomography (CT) data.

**Results:**

Only the visceral-to-subcutaneous fat ratio (*V*/*S* ratio) (cutoff value 0.5) corresponded to a significant difference in OS and PFS in type 1 endometrial cancer (*p* = 0.0080, *p* = 0.0053) according to the results of log-rank tests of Kaplan–Meier curves. The COX regression univariate analysis revealed that only the *V*/*S* ratio was a significant prognostic factor for PFS, but not OS (*p* = 0.033 and *p* = 0.270, respectively).

**Conclusion:**

A *V*/*S* ratio > 0.5 is a possible factor for poor prognosis in type 1 endometrial cancer. Further research is needed to investigate the preventive and therapeutic effects of reducing visceral fat on the prognosis of this type of cancer.

## Introduction

Endometrial cancer is the most common gynecologic cancer. Its incidence is increasing over time and in successive generations in several countries, especially in South Africa and several countries in Asia, including Japan, where rapid socioeconomic transitions are occurring [1, 2]. Endometrial cancer is divided into type 1 and type 2. Type 1 is usually not very aggressive and estrogen dependent, whereas type 2 is more aggressive than type 1 and consists of all other forms of endometrial cancer that do not fall under type 1. The relation between obesity and the development of endometrial cancer is reported to be caused by unopposed estrogen. Estrogen is generated from testosterone by aromatase in adipose cells, which makes obesity a risk factor for type 1 endometrial cancer [3]. On the other hand, the levels of sex hormones are not different between type 1 and type 2, suggesting that type 2 is related to estrogen [4].

Obesity is associated with the incidence of malignant disease [5–7]. Several parameters are used to evaluate obesity, including the body mass index (BMI), abdominal circumference (AC), and visceral-to-subcutaneous fat ratio (*V*/*S* ratio), among others [2]. In particular, visceral fat is an important risk factor for the morbidity and mortality of several cancers [8], and the incidence of endometrial cancer has increased with the occurrence of the obesity epidemic [9].

Visceral obesity measured by ultrasonography is strongly related to the incidence of endometrial cancer [10]. Obesity could possibly influence the prognosis and incidence of endometrial cancer, but information about its impact on the prognosis is limited [11]. Thus, this study investigates the association between prognostic factors of type 1 and type 2 endometrial cancer and obesity parameters, especially the *V*/*S* ratio.

## Materials and methods

### Study design

This retrospective study was approved by the ethics committee of National Hospital Organization Kyoto Medical Center. Institutional Review Board number is 17-061. We searched the pathological database system of the medical center to identify patients who had endometrial cancer from January 2012 to December 2016. The eligibility criterion was a diagnosis of primary uterine endometrial cancer that had been confirmed pathologically. The exclusion criteria were (1) recurrent disease, (2) no surgery, chemotherapy, or radiation therapy, and (3) a lack of computed tomography (CT) images at diagnosis. Ultimately, 145 primary endometrial cancer patients satisfied the eligibility criteria and were enrolled.

The clinical factors examined included age, BMI, pathological findings, Federation of Gynecology and Obstetrics (FIGO) stage, status of lymph node metastasis, amounts of visceral and subcutaneous fat obtained from CT data, progression-free survival (PFS), and overall survival (OS). FATSCAN^®^ was used for the analysis of visceral and subcutaneous fat. CT images at the level of the umbilicus were analyzed to measure visceral fat, subcutaneous fat, and abdominal circumference (AC). The *V*/*S* ratio was calculated to evaluate visceral fat obesity.

### Statistical analyses

The association between two parameters was evaluated by the Mann–Whitney *U* test, while the chi-squared test was used for trend analysis of the contingency table of FIGO stages. PFS was calculated as the time in which the patient was free of cancer after a particular treatment, while OS was calculated as the duration from the date of the first treatment until the date of death or the last follow-up. The Kaplan–Meier method was used to analyze PFS and OS. We used a log-rank test and COX-regression univariate analysis to assess the association between clinical factors and prognosis. The statistical analyses were performed in SPSS (Version 25.0, Inc., Chicago, IL, USA), and *p* < 0.05 was considered statistically significant.

## Results

### Patient characteristics

Table 1 shows the characteristics of endometrial cancer patients. There were 96 cases of type 1 and 52 cases of type 2. The age of the type 1 cases was significantly lower than that of type 2 patients (median ± standard deviation (SD) 55.5 ± 12.5 versus 66.0 ± 9.8 years old; *p* < 0.05). Type 2 cases showed a significantly higher rate of positive results for ascites cytology, lymph vascular involvement, lymph node metastasis, recurrence, and death (*p* < 0.05 for all). There were no statistically significant differences between type 1 and type 2 patients in terms of BMI, AC, total fat area, visceral fat area, subcutaneous fat area, *V*/*S* ratio, familial history of malignancy complication with diabetes mellitus, and complication with hypertension (*p* = 0.41, 0.65, 0.33, 0.16, 0.49, 0.28, 0.85, 0.58, and 1.00, respectively). There were no cases that died from these complications.

**Table 1:** Characteristics of endometrial cancer patients

	All (*n* = 148)	Type 1 (*n* = 96)	Type 2 (*n* = 52)	*p* value (type 1 vs. 2)
Age of diagnosis (median)	61.5	55.5	66.0	< 0.05*
BMI (kg/(m^2^) (median)	23.5	23.6	23.5	0.41
AC (cm) (median)	84.8	84.8	84.5	0.65
Total fat area (cm^2^) (median)	250.0	255.6	220.9	0.33
Visceral fat area (cm^2^) (median)	72.9	82.1	67.2	0.16
Subcutaneous fat area (cm^2^) (median)	176.62	183.6	138.3	0.49
*V*/*S* ratio (median)	0.38	0.39	0.36	0.28
FIGO stage I	106 (71.6%)	84 (87.5%)	26 (50.0%)	< 0.05*
Stage II	7 (4.7%)	4 (4.2%)	3 (5.8%)	
Stage III	22 (14.9%)	6 (6.3%)	16 (30.8%)	
Stage IV	10 (6.8%)	2 (2.1%)	8 (15.4%)	
Positive ascites cytology	20/137 (14.6%)	8/91 (8.8%)	12/46 (26.1%)	< 0.05*
Lymph vascular space invasion	30/109 (27.5%)	11/76(14.5%)	19/36(52.8%)	< 0.05*
Lymph node metastasis	20/127 (15.7%)	7/84 (8.3%)	13/43 (30.2%)	< 0.05*
Recurrence	28/148 (18.96%)	6/96(6.3%)	22/52(42.3%)	< 0.05*
Death	21/148 (14.2%)	4/96(4.2%)	17/52(32.7%)	< 0.05*
Familial history of malignancy	45/148(30.4%)	30/96(31.3%)	15/52(28.8%)	0.85
Complication with diabetes mellitus	29/134 (21.6%)	20/90 (22.2%)	9/44 (20.5%)	0.58
Complication with hypertension	49/139 (35.3%)	34/91 (37.4%)	15/48 (31.3%)	1.00

*Statistical significance (*p* < 0.05)

### Prognostic impacts of obesity parameters

**Fig. 1:**
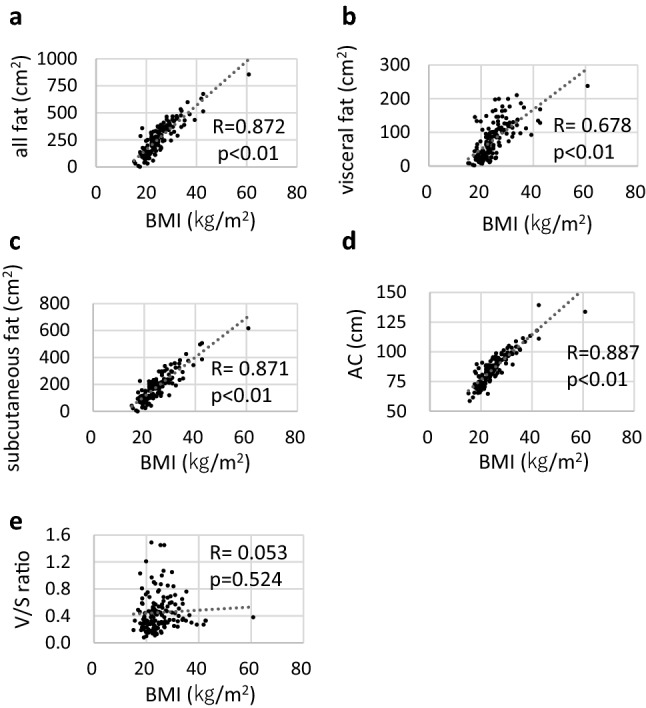
Scatter diagrams of obesity parameters. BMI shows positive correlations with all fat (**a**), visceral fat (**b**), subcutaneous fat (**c**), and AC (**d**), but not with *V*/*S* ratio (**e**)

Figure 1 shows a scatter diagram of the obesity parameters. We analyzed the association between BMI and other obesity parameters. There was a strong correlation between BMI and all types of fat (*R* = 0.87, *p* < 0.01; Fig. 1a), visceral fat (*R* = 0.67, *p* < 0.01; Fig. 1b), subcutaneous fat (*R* = 0.87, *p* < 0.01; Fig. 1c), and AC (*R* = 0.88, *p* < 0.01; Fig. 1d). However, BMI and *V*/*S* ratio did not show a significant correlation (*R* = 0.05, *p* = 0.52; Fig. 1e).

**Fig. 2:**
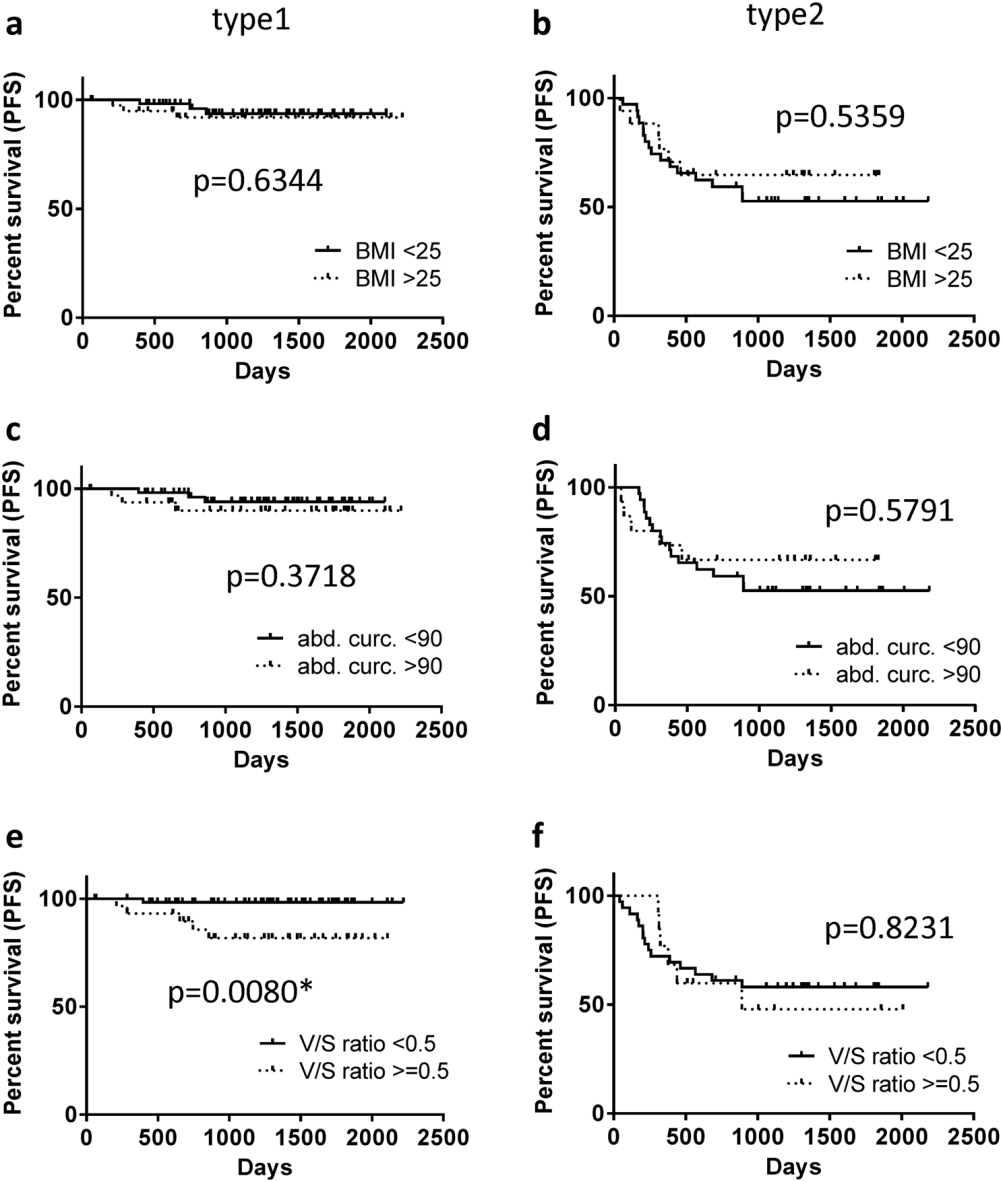
Kaplan–Meier curves of type 1 and type 2 endometrial cancers for PFS. Log-rank tests indicate that BMI and AC are not significant prognostic factors in both type 1 and 2 endometrial cancers (**a**–**d**), whereas the group with a high *V*/*S* ratio shows poorer prognosis than the one with a low *V*/*S* ratio in type 1 endometrial cancer (**e**), but not in type 2 endometrial cancer (**f**)

**Fig. 3:**
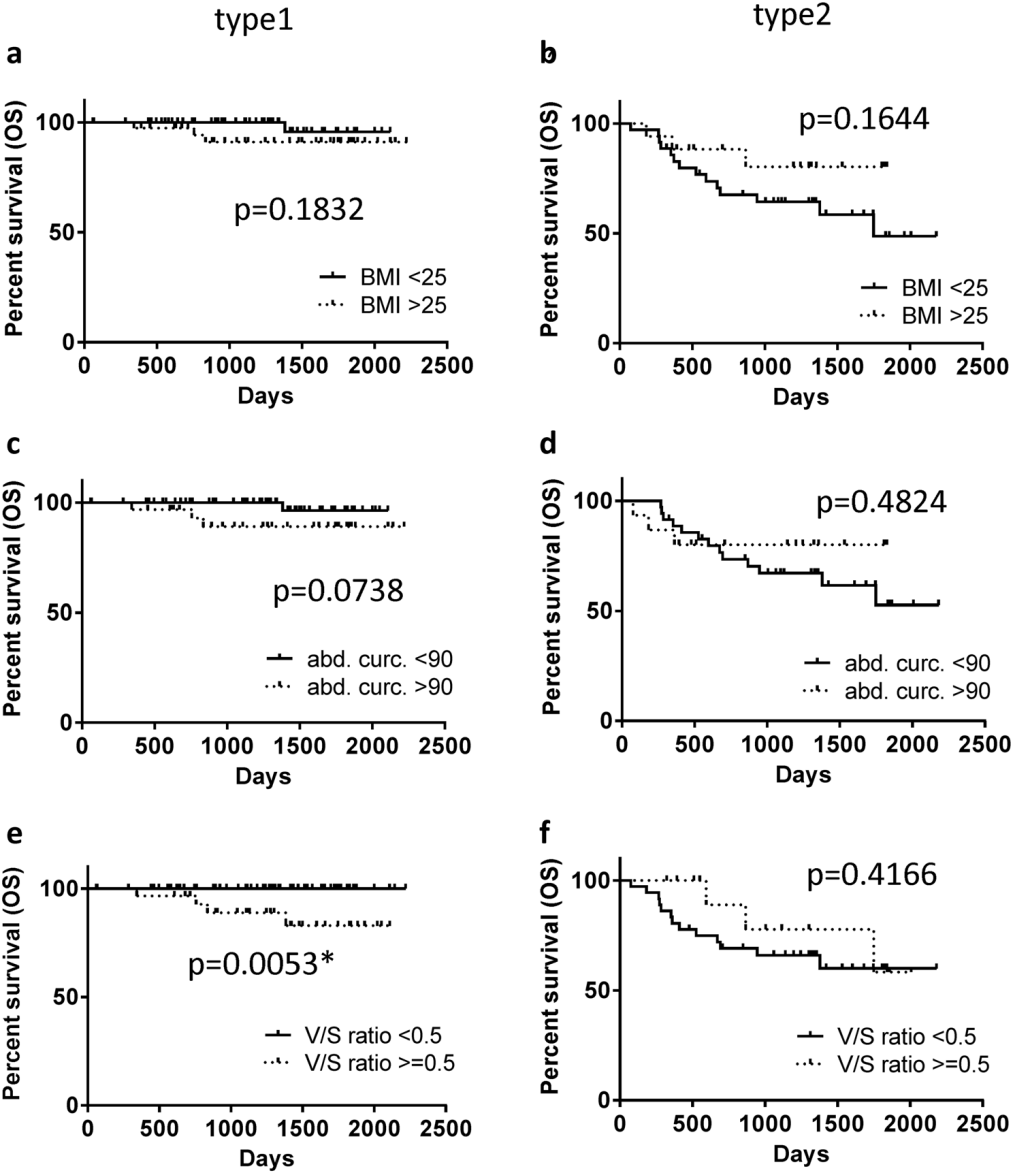
Kaplan–Meier curves of type 1 and type 2 endometrial cancers for OS. Log-rank tests indicate that BMI and AC are not significant prognostic factors in both type 1 and 2 endometrial cancers (**a**–**d**). Those with high *V*/*S* ratio shows poorer prognosis than those with low *V*/*S* ratio in type 1 endometrial cancer (**e**), but not in type 2 endometrial cancer (**f**)

We performed log-rank tests on OS and PFS for each type of endometrial cancer in terms of BMI (cutoff value 25 kg/m^2^; Figs. 2a, b and 3a, b; PFS and OS, respectively), AC (cutoff value 100 cm; Figs. 2c, d and 3c, d; PFS and OS, respectively), and *V*/*S* ratio (cutoff value 0.5; Fig. 2e, f, 3e, f; PFS and OS, respectively). For PFS, Kaplan–Meier curves of type 1 and type 2 endometrial cancer showed no significant difference between BMI > 25 kg/m^2^ and < 25 kg/m^2^ (*p* = 0.6344 and *p* = 0.5359, respectively; Fig. 2a, b). AC showed no significant difference between AC ≥ 100 cm and < 100 cm in both type 1 and type 2 endometrial cancers (*p* = 0.3718 and *p* = 0.5791, respectively; Fig. 2c, d). However, Kaplan–Meier curves of the *V*/*S* ratio identified a significant difference between *V*/*S* ratios > 0.5 and < 0.5 in type 1 endometrial cancer, but not in type 2 *p* = 0.0080 and *p* = 0.8231, respectively; Fig. 2e, f.

For OS, BMI and AC did not show statistically significant differences for type 1 and type 2 (*p* = 0.1832, *p* = 0.1644 and *p* = 0.0738 and *p* = 0.4824, respectively; Fig. 3a–d). However, the *V*/*S* ratio showed a statistically significant difference for type 1 but not type 2 (*p* = 0.0053 and *p* = 0.4166, respectively; Fig. 3e, f). We also analyzed obesity parameters using a COX regression univariate analysis of PFS and OS. For PFS, only the *V/S* ratio was a significant prognostic factor (*p* = 0.033, Table 2), whereas there were no significant prognostic factors for OS (Table 3).

**Table 2:** COX regression univariate analysis of progression-free survival in type 1 endometrial cancer patients

	PFS	Crude odds ratio	95% CI		*p* value
			Lower	Upper	
Age	≦ 50, *n* = 33 > 50, *n* = 63	40.171	0.037	43,957.868	0.301
BMI	< 25, *n* = 56 ≧ 25, *n* = 40	1.471	0.297	7.290	0.636
Abd. Circ	< 90, *n* = 58 ≧ 90, *n* = 33	1.929	0.421	0.421	0.421
*V*/*S* ratio	< 0.5, *n* = 30 ≧ 0.5, *n* = 61	10.343	1.208	88.558	0.033*
FIGO stage	I, *n* = 84 II–IV, *n* = 12	3.590	0.657	19.623	0.140
LVSI	Absent, *n* = 65 Present, *n* = 11	6.746	0.422	107.959	0.177
Ascites	Negative, *n* = 83 Positive, *n* = 8	0.042	0.000	4389.474	0.591
LNs metastasis	Absent, *n* = 78 Present, *n* = 6	3.218	0.375	27.589	0.286

*Statistical significance (*p* < 0.05)

**Table 3:** COX regression univariate analysis of overall survival in type 1 endometrial cancer patients

	PFS	Crude odds ratio	95% CI		*p* value
			lower	upper	
Age	≦ 50, *n* = 33 > 50, *n* = 63	38.504	0.006	230,030.880	0.411
BMI	< 25, *n* = 56 ≧ 25, *n* = 40	4.127	0.428	39.757	0.220
Abd. Circ	< 90, *n* = 58 ≧ 90, *n* = 33	5.687	0.591	54.678	0.132
*V*/*S* ratio	< 0.5, *n* = 30 ≧ 0.5, *n* = 61	160.894	0.019	1,331,706.347	0.270
FIGO stage	I, *n* = 84 II–IV, *n* = 12	6.284	0.875	45.109	0.068
LVSI	Absent, *n* = 65 Present, *n* = 11	6.573	0.411	105.146	0.183
Ascites	Negative, *n* = 83 Positive, *n* = 8	0.041	0.000	25,209.750	0.638
LNs metastasis	Absent, *n* = 78 Present, *n* = 6	4.464	0.460	43.344	0.197

*Statistical significance (*p* < 0.05)

## Discussion

The results revealed that *V*/*S* ratio > 0.5 was a factor for poorer prognosis in cases of type 1 endometrial cancer. BMI is a major parameter of obesity, and although it is known to be risk factor for endometrial cancer [5–7], its relation with the prognosis has not been investigated sufficiently [11]. Obesity is divided into visceral-type obesity and subcutaneous-type obesity. The visceral type is more severe than the subcutaneous type because of its higher incidence of obesity-related diseases, such as diabetes mellitus, cerebrovascular disease, and ischemic heart disease [8].

To our knowledge, this is the first report to indicate that the *V*/*S* ratio is related to OS and PFS of type 1 endometrial cancer. However, three previous studies have examined the *V*/*S* ratio and prognostic factors in endometrial cancer. Ye et al. reported that in all types of endometrial cancer, 31.89% visceral adiposity (VAT%) (equivalent to a *V*/*S* ratio of 0.46) was a cutoff value for a significant difference in lymph node metastasis and extrauterine disease. They designated the median value of their study as the cutoff value. However, prognostic factors such as OS or PFS were not investigated [12].

Another study also used the median value of their study as a cutoff value. They found that VAT% greater than 37% (equal to a *V*/*S* ratio of 0.58) showed a negative prognostic impact on both PFS and OS in all types of endometrial cancer [13]. Cases with high visceral adiposity had higher mortality because of endometrial cancer [14]. However, the studies did not identify the difference between type 1 and type 2 endometrial cancers. Resent study identified that percentage of visceral out of total abdominal fat volume is associated with poor overall- and disease-specific survival in non-endometrioid endometrial cancer patients [15]. They did not use the *V/S* ratio and not focused on type 1 endometrial cancer.

One report indicates that estradiol levels were not different regardless of menopausal status in type 1 and type 2 endometrial cancer [4]. However, our findings suggest that visceral fat is related to poor prognosis in only type 1 endometrial cancer, which is known to be estrogen dependent. Furthermore, a *V*/*S* ratio of more than 0.4 is reported as a cutoff value for visceral-type obesity for cardiovascular disease [16]. In endometrial cancer, values of the *V*/*S* ratio that are related to prognosis are higher than the *V*/*S* ratio of 0.4 for detecting the risk of obesity-related diseases such as diabetes mellitus. This suggests that severe visceral-type obesity is a possible prognostic factor for endometrial cancer.

This study has some limitations. If the patients did not receive any therapies, they were excluded from this study, which means that it could be possible that the most advanced cases were not assessed. We did not perform lymphadenectomy in advanced stages. It should affect the prognosis. Furthermore, the Cox regression models did not show any statistical significance of the impact of poor clinical factors, including stage, ascites cytology, lymph vascular involvement, and lymph node metastasis. The possible reasons include a limited number of type 1 endometrial cancer patients, which show a favorable prognosis. We did not measured the serum levels of estradiol. Lastly, we did not validate the findings using another dataset.

In conclusion, a *V*/*S* ratio > 0.5 is a possible factor for poor prognosis in type 1 endometrial cancer. Nevertheless, further research is needed to investigate the preventive and therapeutic effects of the reduction of visceral fat on the prognosis of this type of cancer.
